# Anti-Glide Plate Fixation for Lateral Malleolus Fractures by Minimally Invasive Technique in Geriatric Patients

**DOI:** 10.7759/cureus.23160

**Published:** 2022-03-14

**Authors:** Shamunyama Mooya, Muhammad Kakakhel, Ahmed El-Amien, Padinjarathala Anto

**Affiliations:** 1 Trauma and Orthopaedics, University Hospital Waterford, Waterford, IRL

**Keywords:** orthogeriatric medicine, lateral malleolus, short musculoskeletal function assessment (smfa), american orthopaedic foot and ankle society 24 (aofas), elderly, orif, anti-glide, ankle fracture

## Abstract

The objective was to evaluate the functional outcomes of anti-glide fixation by minimally invasive plate osteosynthesis (MIPPO) in lateral malleolus ankle fractures.

The study was a retrospective cohort study conducted at a single trauma center. We reviewed 39 patients >60 years old with either isolated or non-isolated lateral malleolus Weber B ankle fractures.

The main outcome measures were postoperative functional assessment performed with the American Orthopaedic Foot and Ankle Society (AOFAS) and Short Musculoskeletal Functional Assessment (SMFA) scores. Our results showed that the mean time to surgery was 1 day. Seventeen patients underwent surgery within 24 hours after sustaining the injury. The mean AOFAS and SMFA scores were 87.8 and 209.7, respectively. No patient developed implant failure or wound complications.

It was concluded that the anti-glide plating of the lateral malleolus had better functional outcomes compared to lateral plating by open reduction and internal fixation (ORIF), as shown by the higher AOFAS scores and fewer postoperative complications.

## Introduction

Ankle fractures are the third most common fracture in the elderly population, following hip and distal radius fractures [1]. Annually, 100 to 200 ankle fractures per 100,000 individuals are reported [2-3], among this, 20% to 30% occur in the elderly [4]. Traditionally, elderly and medically complex patients were treated conservatively. However, the increased morbidity with non-operative management has led to the development of operative techniques with ideal outcomes in elderly patients and those with significant comorbidities.

Lateral malleolus fractures most commonly result from supination-external rotation injuries, in a common posterosuperior to inferoanterior direction. These are classified as Danis-Weber B fractures, which can be isolated or involve the medial malleolus. Fixation can be achieved in a variety of ways: direct open reduction and internal fixation with lag screw fixation and neutralization plating (lateral or posterolateral) or bridge plating (lateral or posterolateral), using a 1/3 tubular plate or precontoured locking plate, or intramedullary fixation with a screw or nail (i.e., Fibulock).

This retrospective cohort study aimed to evaluate the functional outcomes of posterolateral anti-glide fixation using minimally invasive plate osteosynthesis (MIPPO). Notably, the study’s population included bi- and trimalleolar fractures in addition to isolated malleolar fractures. Patients with suspected syndesmotic injury were assessed intraoperatively and managed through fixation using syndesmotic screws.

## Materials and methods

This retrospective cohort study included patients who underwent anti-glide plate fixation of the lateral malleolus at a trauma center in the southeast region of Ireland. Data was collected between May 2012 and November 2020. This study included patients with Weber B ankle fractures with additional medial or posterior malleolus fractures, or those with concomitant syndesmosis injury. Patients with open fractures or those who were not classified as Weber B were excluded from the study. Patients were divided and examined using cohorts based on factors that influence functional outcomes and wound healing, such as age, diabetes, and the presence of syndesmotic screw fixation. Functional outcomes were measured using the American Orthopaedic Foot and Ankle Society (AOFAS) ankle-hindfoot scale and the Short Musculoskeletal Function Assessment (SMFA). Complications were also noted. Due to the growing geriatric population in Ireland and the world, our study focused on patients over the age of 60 as the observed cohorts.

All surgical operations were performed by a senior consultant surgeon under similar conditions. The patients were placed in a supine position on a radiolucent table, and a tourniquet with a sandbag was applied to the hip of the affected side to provide tilt and help with visualisation. A 3- to 4-cm posterolateral longitudinal incision was made extending from the apex of the fracture proximally; subsequently, the fibula was indirectly reduced. The periosteum was elevated, and a one-third tubular plate was used for fixation. Plate application is achieved by initially sliding the plate proximally into the incision and then distally with screening under an image intensifier to ensure appropriate positioning. The plates utilised were titanium 1/3 tubular plates (Synthes, Switzerland) with a minimum length of five holes; these were secured with three proximal cortical screws in an anti-glide manner. The lateral malleolus was fixed first to regain length and facilitate anatomic repositioning of the talus in the ankle mortise. Patients with bimalleolar fractures required fixation of the medial malleolus, with insertion of a syndesmosis screw, if necessary. Syndesmotic injury was intraoperatively assessed in four patients after internal fixation of both the malleoli by using a bone hook to lever the fibula laterally to check for instability. A long 3.5-mm cortical screw was passed through the fibular plate from a posterolateral to anteromedial direction.

Postoperatively, a walker boot was used to immobilize the ankle for six weeks. The patients were followed up at two and six weeks postoperatively. Partial and full weight-bearing were allowed at three and six weeks, respectively.

Surgical outcomes were evaluated clinically using functional assessment scores, such as the American Orthopaedic Foot and Ankle Society (AOFAS) Ankle-Hindfoot Scale and Short Musculoskeletal Function Assessment (SMFA). The AOFAS score is used in patients with an ankle or hindfoot injury to assess functional outcomes and pain, critical factors for patient recovery [5-6]. It is one of the commonly used outcome measurement tools by foot and ankle specialists to determine patient limitations postoperatively [7]. A score of 90 to 100 is excellent; 80 to 89, good; 70 to 79, fair; and below 69, poor. On the other hand, the Musculoskeletal Function Assessment (MFA) is a 101-item questionnaire used to assess patients with a wide variety of common musculoskeletal disorders of the arms and legs [8]. The SMFA questionnaire is a 46-item shortened version of the MFA questionnaire and includes items covering dysfunction [9]. The SMFA consists of two sections: functional assessment and degree of patient discomfort with their symptoms, which have 34 and 12 questions, respectively. SMFA is not widely used; however, its questionnaire format enables easier implementation.

## Results

A total of 106 patients who underwent anti-glide plate fixation of the lateral malleolus were included in this study; among them, 39 (36.8%) were >60 years old. Additionally, 61 (57.5%) participants were female and 45 (42.5%) were male (Table 1). Patients were followed up at two weeks, six weeks, and three months postoperatively. Among the 39 elderly patients, 28 were assessed using SMFA and AOFAS with a mean score of 209.7 (Table 2) and 87.8 (Table 3), respectively. No patient developed implant failure or wound complications. Additionally, we achieved a 100% fracture union rate at the final follow-up of three months. A modified RUST (mRUST) score [10] and clinical assessment [11] were used to determine the union.

**Table 1:** Patient demographics AOFAS = The American Orthopaedic Foot and Ankle Society score SMFA = Short Musculoskeletal Function Assessment score

Age > 60	39
Male	45
Female	61
AOFAS	28
SMFA	28

**Table 2:** AOFAS score AOFAS = The American Orthopaedic Foot and Ankle Society score

	AOFAS scores
Mean	87.8
Mode	79

**Table 3:** SMFA score SMFA = Short Musculoskeletal Function Assessment score

	SMFA scores
Mean	209.7
Mode	179

In 39 patients, the mean time to surgery was 1 day (Table 4), and the total number of days to surgery was 102 days; however, the most common time to surgery (mode) was 1 day. Additionally, 17 patients (49%) underwent surgery within 24 hours after sustaining the injury. The longest time to surgery was 17 days, which is an outlier in our dataset. The patient was referred from a nearby peripheral hospital where this patient was admitted for medical management.

**Table 4:** Clinical and radiological follow-up AOFAS = The American Orthopaedic Foot and Ankle Society score SMFA = Short Musculoskeletal Function Assessment score LOS/days = length of stay in days

	Anti-glide plate
Time to surgery/days	1
LOS/days	2
Time to discharge from surgery/days	1.1
AOFAS	87.8
SMFA	209.7
Union/cases	39
Implant failure/cases	0
Wound failure/cases	0

All patients achieved union at 12 weeks as defined by mRUST score. One patient in our subgroup of interest experienced persistent ankle pain, which necessitated implant removal. This was performed at 20 weeks postoperatively and in the context of pre-existing ankle osteoarthritis.

We were unable to perform follow-up scores on 11 patients with either AOFAS or SMFA; among them, four patients died, and five were lost to follow-up.

## Discussion

Our study showed that anti-glide fixation of lateral malleolus fractures by MIPPO in the geriatric population was beneficial. This was shown by our results: mean time to surgery was 1 day, mean length of stay in hospital was 2 days and time to discharge was just over 1 day. Union was also achieved in all the 39 cases operated on. No surgical complications resulted, such as wound and implant failure.

Previous studies have found significant variations regarding AOFAS scores from lateral malleolar ankle fracture open reduction and internal fixation (ORIF). Functional assessment using AOFAS score has been correlated with several factors including age, comorbidities, and baseline function; however, systematic reviews regarding ankle ORIFs in the elderly remain lacking. Luong et al. [12] found that minimally invasive techniques for the fixation of distal fibular fractures can provide excellent functional results with low complication rates compared with traditional ORIF.

The AOFAS score is an accepted research tool in the assessment of postoperative function and was used to compare our outcomes with those in the published literature. Previous studies have assessed the postoperative function of patients who underwent lateral plate ORIF using AOFAS, which can be compared with this study that investigated anti-glide fixation of the lateral malleolus.

Zhong et al. [13] showed an AOFAS score of 74.5 in patients aged 18-64 years after ORIF. Furthermore, Zhenhua et al. [14] retrospectively reviewed 20 patients with fractures of the distal lateral malleolus and found an AOFAS score of 78 in an age group similar to that in our study. The mean follow-up in this study was 21.4 months (range, 16-27 months). Four patients developed complications, including post-traumatic osteoarthritis, hardware impingement, and superficial wound infection; however, these complications were not correlated with AOFAS score.

Xia et al. [15] found an AOFAS score of 88 in 21 patients with ages ranging from 23 to 69 years, with purely lateral malleolus fractures. Each patient underwent ORIF with a mini-locking plate for internal fixation. It should be noted that compression of the fracture end was performed concomitantly with an arched-shape-memory connector (ASC), which would affect the AOFAS score.

A prospective, randomised study by Salai et al. [16] investigated 84 patients with displaced ankle fractures >65 years; they found that the AOFAS score was significantly higher in the patients conservatively managed compared to patients who underwent operative treatment (91.37±8.96 vs 75.2±14.38).

Finally, the retrospective study of Emara et al. [17] on 26 patients with uncontrolled diabetes found a median AOFAS score of 60. This low AOFAS score may be attributed to three patients (11.5%) who developed complications, including Charcot joint, fracture displacement that needed revision, and malreduction.

A prospective study by Kilian et al. [18] found no significant difference in the AOFAS score of patients who underwent anti-glide (93.7±6.1; range 85-100) or lateral plate fixation of Weber B malleolus fractures (94.5±6.0; range 85-100) (p = 0.37). However, lag screws were applied in some patients who underwent anti-glide operation, which may have confounded the results.

A further advantage of this technique is that it can be performed in patients with a degree of swelling around the ankle. The general approach to such patients using traditional fixation techniques is to elevate the ankle in the ward for several days until swelling subsides, only after ORIF is performed. Using MIPPO, a posterolateral incision can be made, and surgery is performed at an earlier stage, allowing more rapid treatment and discharge. This will lead to a significantly reduced hospital length of stay, which is of particular importance at this time with the prevalence of coronavirus disease in hospitals.

## Conclusions

This study primarily found that anti-glide plating by MIPPO has better functional outcomes than lateral plating by ORIF, as shown by the higher AOFAS scores. Due to the nature of the surgical procedure, there is minimal periosteal stripping and surgical incisions, and there are fewer postoperative complications such as peroneal complications and wound dehiscence.

Further research, ideally by a randomised prospective study, comparing the anti-glide fixation of the lateral Weber B fracture versus lateral plating with locking or one-third tubular plating by ORIF, would validate the results of this study.

## References

[1] Van Lieshout EM, De Boer AS, Meuffels DE, Den Hoed PT, Van der Vlies CH, Tuinebreijer WE, Verhofstad MH (2017). American Orthopaedic Foot and Ankle Society (AOFAS) Ankle-Hindfoot Score: a study protocol for the translation and validation of the Dutch language version. BMJ Open.

[2] Shazadeh Safavi P, Janney C, Jupiter D, Kunzler D, Bui R, Panchbhavi VK (2019). A systematic review of the outcome evaluation tools for the foot and ankle. Foot Ankle Spec.

[3] Jensen SL, Andresen BK, Mencke S, Nielsen PT (1998). Epidemiology of ankle fractures. A prospective population-based study of 212 cases in Aalborg, Denmark. Acta Orthop Scand.

[4] Court-Brown CM, Caesar B (2006). Epidemiology of adult fractures: a review. Injury.

[5] Malviya A, Makwana N, Laing P (2018). AOFAS scores. Trend and correlation with QALY score. Bone & Joint J.

[6] Ehrenfreund T, Haluzan D, Dobric I, Zigman T, Rajacic D, Antoljak T, Davila S (2013). Operative management of unstable ankle fractures in the elderly: our institutional experience. Injury.

[7] Kadakia RJ, Ahearn BM, Schwartz AM, Tenenbaum S, Bariteau JT (2017). Ankle fractures in the elderly: risks and management challenges. Orthop Res Rev.

[8] Engelberg R, Martin DP, Agel J, Obremsky W, Coronado G, Swiontkowski MF (1996). Musculoskeletal function assessment instrument: criterion and construct validity. J Orthop Res.

[9] Swiontkowski MF, Engelberg R, Martin DP, Agel J (1999). Short musculoskeletal function assessment questionnaire: validity, reliability, and responsiveness. J Bone Joint Surg Am.

[10] Litrenta J, Tornetta P 3rd, Mehta S (2015). Determination of radiographic healing: an assessment of consistency using RUST and modified RUST in metadiaphyseal fractures. J Orthop Trauma.

[11] Morshed S (2014). Current options for determining fracture union. Adv Med.

[12] Luong K, Huchital MJ, Saleh AM, Subik M (2021). Management of distal fibular fractures with minimally invasive technique: a systematic review. J Foot Ankle Surg.

[13] Zhong S, Shen L, Zhao JG (2017). Comparison of posteromedial versus posterolateral approach for posterior malleolus fixation in trimalleolar ankle fractures. Orthop Surg.

[14] Zhenhua F, Waizy H, Ming X, Wusheng K (2013). Lateral malleolus hook plate for comminuted Weber A and B fractures: a retrospective study. Indian J Orthop.

[15] Xia D, Zhang Y, Ou T, Wang Y, Hao Z, Zhou P, Xu S (2020). Combination of mini locking plate and nitinol arched shape-memory connector for purely lateral malleolus fractures: technique and clinical results. Ann Transl Med.

[16] Salai M, Dudkiewicz I, Novikov I, Amit Y, Chechick A (2000). The epidemic of ankle fractures in the elderly--is surgical treatment warranted?. Arch Orthop Trauma Surg.

[17] Emara KM, Diab RA, Essa MN, Gemeah M, Emara YK, Fleifil S (2020). Percutaneous cannulated screw fixation in the treatment for diabetic ankle fractures. Eur J Orthop Surg Traumatol.

[18] Kilian M, Csörgö P, Vajczikova S, Luha J, Zamborsky R (2017). Antiglide versus lateral plate fixation for Danis-Weber type B malleolar fractures caused by supination-external rotation injury. J Clin Orthop Trauma.

